# Unlocking the potential of stimuli-responsive biomaterials for bone regeneration

**DOI:** 10.3389/fphar.2024.1437457

**Published:** 2024-07-31

**Authors:** Ke Yang, Zhuoshu Wu, Keke Zhang, Michael D. Weir, Hockin H. K. Xu, Lei Cheng, Xiaojing Huang, Wen Zhou

**Affiliations:** ^1^ Fujian Key Laboratory of Oral Diseases & Fujian Provincial Engineering Research Center of Oral Biomaterial & Stomatological Key Lab of Fujian College and University, School and Hospital of Stomatology, Fujian Medical University, Fuzhou, China; ^2^ Clinical Research Center for Oral Tissue Deficiency Diseases of Fujian Province, School and Hospital of Stomatology, Fujian Medical University, Fuzhou, China; ^3^ School and Hospital of Stomatology, Wenzhou Medical University, Wenzhou, China; ^4^ Department of Biomaterials and Regenerative Dental Medicine, University of Maryland School of Dentistry, Baltimore, MD, United States; ^5^ State Key Laboratory of Oral Diseases & National Clinical Research Center for Oral Diseases, West China School of Stomatology & Department of Operative Dentistry and Endodontics, West China Hospital of Stomatology, Sichuan University, Chengdu, China

**Keywords:** bone regeneration, stimuli-responsive biomaterials, scaffolds, implants, composites, hydrogels

## Abstract

Bone defects caused by tumors, osteoarthritis, and osteoporosis attract great attention. Because of outstanding biocompatibility, osteogenesis promotion, and less secondary infection incidence ratio, stimuli-responsive biomaterials are increasingly used to manage this issue. These biomaterials respond to certain stimuli, changing their mechanical properties, shape, or drug release rate accordingly. Thereafter, the activated materials exert instructive or triggering effects on cells and tissues, match the properties of the original bone tissues, establish tight connection with ambient hard tissue, and provide suitable mechanical strength. In this review, basic definitions of different categories of stimuli-responsive biomaterials are presented. Moreover, possible mechanisms, advanced studies, and pros and cons of each classification are discussed and analyzed. This review aims to provide an outlook on the future developments in stimuli-responsive biomaterials.

## Graphical Abstract

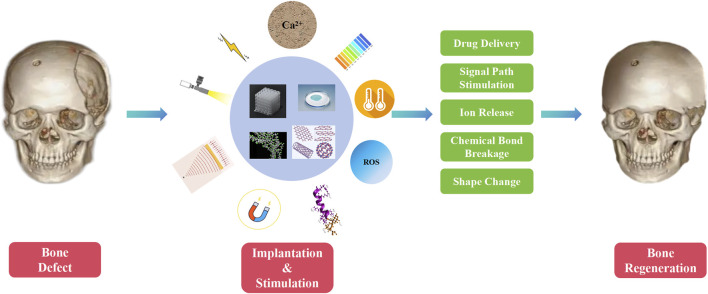

## 1 Introduction

Worldwide incidences of bone defects and other bone-related complications, including bone infection, bone tumor, and bone loss, have increased owing to the growing life expectancy and the aging population (Bose et al., 2023). Among all clinical available bone grafts, autogenous ones are the gold standard (Wang and Yeung, 2017). However, deficient blood supply and morbidity of donor site complications are the main limitations. Though allografts take up the second higher option, they also have several drawbacks, including secondary injury, limited resources, risk of infectious disease, and immunological rejection (De Long et al., 2007; Wei et al., 2022a). Moreover, various new bone restorative strategies have emerged, which comprise tissue-engineered periosteum and platelet-rich plasma (Fang et al., 2020; Zhang et al. (2022b). Nevertheless, because of the inadequate clinical application, their capacity for hard-tissue regeneration and drug delivery precision should be further verified (Bose et al., 2019; Fan et al., 2021; Losic, 2021). Hence, investigations on more intelligent bone grafting materials for treating bone disorders and reconstructing bone defects have radically become the focus in this field.

Bone tissue engineering (BTE) is a novel tactic for promoting bone regeneration by combining biomaterials and bioactive factors to encourage cells to migrate, attach and proliferate (Shang et al., 2021). Consequently, ideal restorative biomaterials should perform good mechanical strength, biocompatibility and steadily controllable drug release ability (Handa et al., 2022; Ni et al., 2023). Stimuli-responsive biomaterials are newly developed alternative materials for BTE, opening new frontiers in the development of scaffolds and implants. These biomaterials can mimic the dynamic nature of the native extracellular matrix, providing a more conducive environment for cell growth, differentiation, and bone regeneration. Therefore, stimuli-responsive biomaterials occupy a critical position in the development of restorative bone grafts.

Stimuli-responsive biomaterials for BTE include scaffolds, nanoparticles, biopolymers, hydrogel and so on. When these materials are exposed to certain stimuli, their mechanical properties, shape, form or drug-releasing rate may change accordingly to match the properties of the original bone tissues, establish tight connection with ambient hard tissue, and provide adequate mechanical strength (Lin et al., 2013; Islam et al., 2020; Sadowska and Ginebra, 2020; Wang et al., 2020; Shang et al., 2021). These materials are promising in clinical application. For instance, stimuli-responsive biomaterials like pH-responsive chitosan (CS) have been widely utilized in bone regeneration (Arora et al., 2023).

**FIGURE 1:**
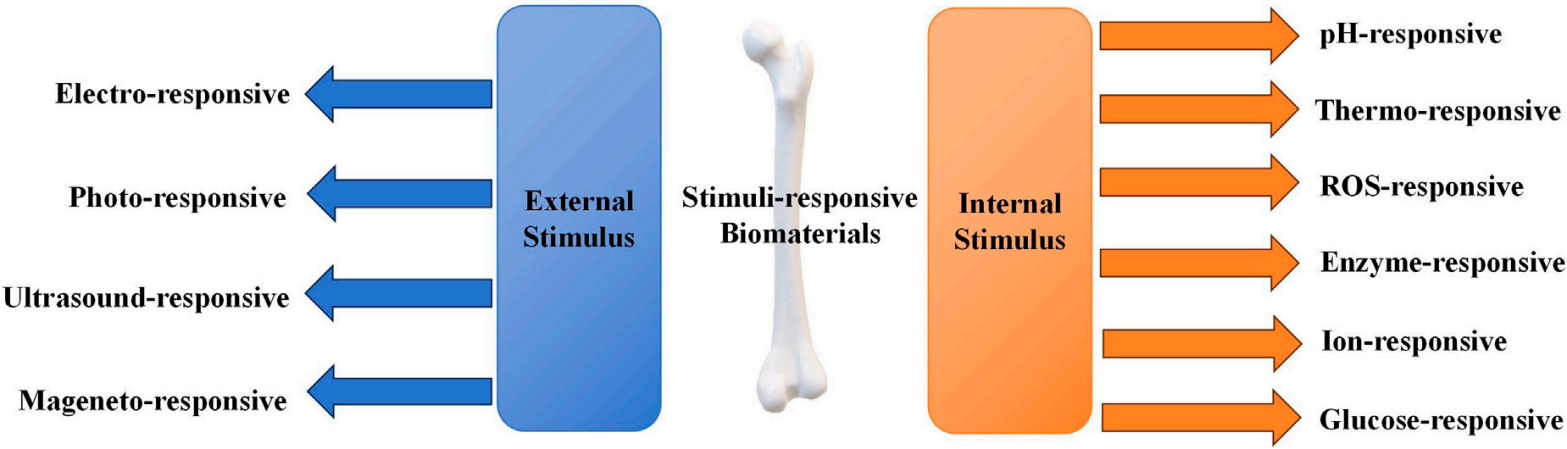
Categories of stimuli-responsive biomaterials.

Stimuli-responsive biomaterials can be categorized into two main types: 1) external stimuli-responsive biomaterials; 2) internal stimuli-responsive biomaterials (Figure 1) (Wei et al., 2022a). External stimuli-responsive biomaterials include implants that can change their properties after being activated by external stimuli. The activated materials can affect the cell fate and enhance bone tissue regeneration by altering the mechanical characteristics, breaking chemical bonds, or causing other transformations (Lui et al., 2019). On the contrary, internal stimuli-responsive biomaterials are the ones that can respond to specific microenvironmental changes surrounding the lesion, such as decrease in pH, alteration of temperature, increase in reactive oxygen species (ROS), and fluctuation in enzyme levels (Du et al., 2019). Recently, stimuli-responsive biomaterials have been widely studied in controlling the release of loaded drugs (Yadav et al., 2023). By specific stimuli activating, changes are triggered in the stimuli-responsive biomaterials, which include self-assembly of peptides, breakage of chemical bonds, and alteration in release behaviors (Li et al., 2024; Mu et al., 2024). Thus, the activated materials can modulate certain cellular pathways related to the induction of bone regeneration.

In this review, recent studies on the development and mechanism of stimuli-responsive biomaterials in bone regeneration are summarized. An outlook on future advancements in stimuli-responsive biomaterials has also been provided.

## 2 Biomaterials responding to external stimuli

**FIGURE 2:**
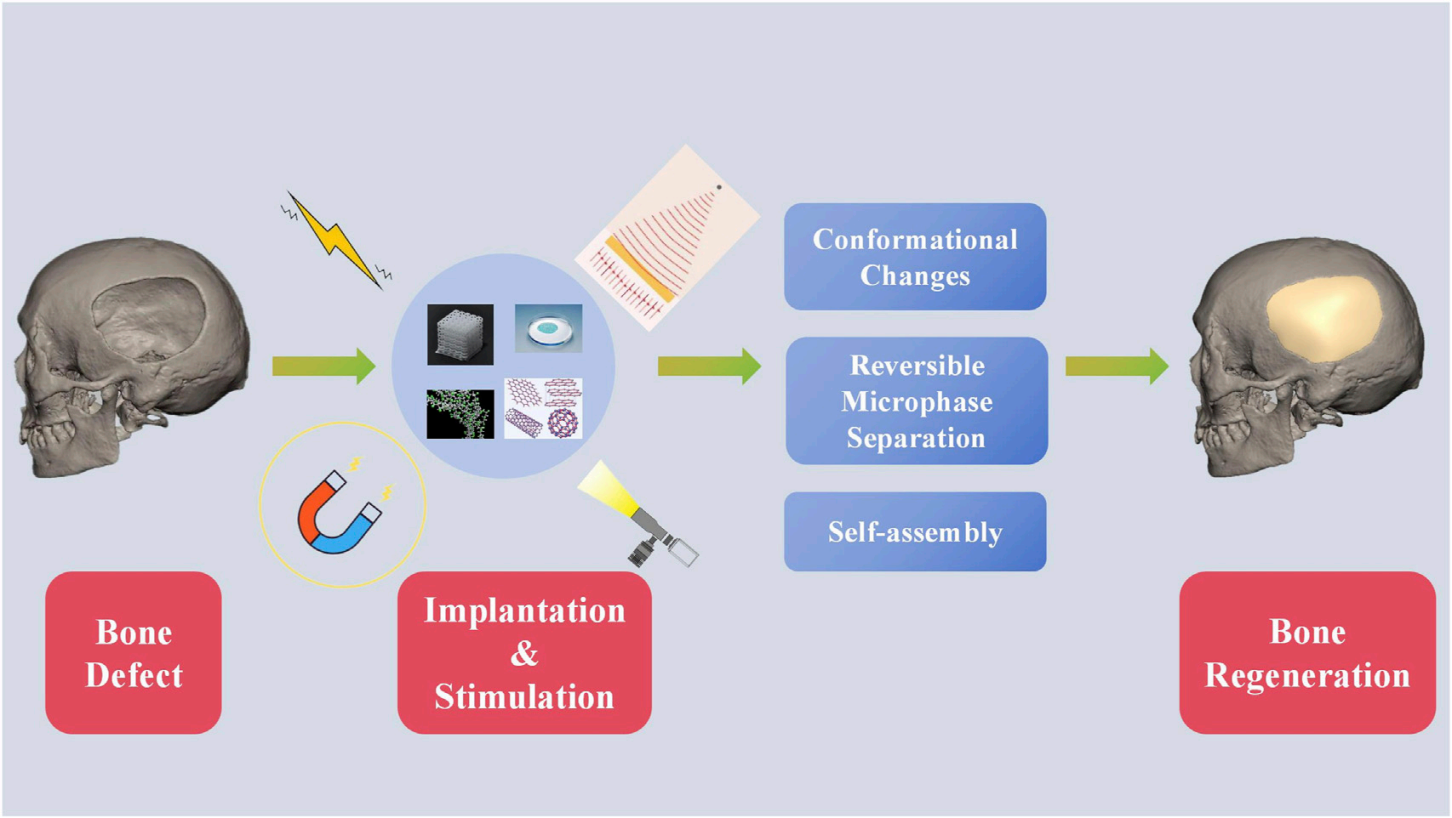
Schematics of external stimuli-responsive biomaterials for bone regeneration.

External or out-body stimuli are signals or irradiations applied outside the body (Fang et al., 2021; Montoya et al., 2021). External stimuli include electricity, light, ultrasound, and magnetic field. External stimuli-responsive biomaterials undergoes conformational changes, reversible microphase separation, or self-assembly according to the stimulus (Figure 2) (Montoya et al., 2021). Subsequently, the activated materials can affect cell attachment, migration, and proliferation to enhance osteogenesis (Wei et al., 2022a).

### 2.1 Electro-responsive composites

Electrical stimulation (EStim) can improve bone regeneration by promoting bone-forming stem cells activities like migration, proliferation, differentiation, mineralization, extracellular matrix deposition, and attachment to scaffold materials (George et al., 2017; Leppik et al., 2020). Generally, there are two kinds of EStim, direct (external electric field) and indirect ones (piezoelectrical field) (Zhu et al., 2023).

When exposed to the certain external electric stimulator, electro-responsive composites can deliver localized electrical signals (Wei et al., 2022b). Followingly, these signals can stimulate the calcium–calmodulin pathway or induce the transformation of other cytokines, which form the basis of osteogenesis (Wei et al., 2022b). Under exogenous electrical stimulation, the delivered electrical signals can contribute to upregulating level of transforming growth factor-β (TGF-β), bone morphogenetic proteins-2 (BMP-2) and the expression of Runt-related transcription factor 2 (Runx2), Osteocalcin (OCN), and Osteopontin (OPN) (Fu et al., 2019; Khare et al., 2020; Wei et al., 2022b).

Zhang et al. prepared polypyrrole (PPY) nanoparticles dispersed in a chitosan matrix with BMP-2 covalently immobilized on the surface (PPY/chitosan films) (Zhang et al., 2013). When osteoblasts cultured on the electrical stimulated PPY/chitosan film, greater increasing in cellular metabolic activity, ALP activity and mineralization over control group were observed (Zhang et al., 2013). Moreover, osteogenic gene expression analysis showed that releasing of BMP-2 and exogenous electrical stimulation did have synergistic effects on osteoblast differentiation and maturation (Zhang et al., 2013).

Piezoelectric scaffolds are biomaterials with a uniquely porous morphology (Sultana et al., 2017; Chorsi et al., 2019; Liu et al., 2023). Under mechanical stress, transient deformation of piezoelectric scaffold occurs, leading to an atomic position shift (Mao et al., 2022). This phenomenon results in the loss of the center of symmetry and induces the accumulation of charge through an ordered dipole distribution (Mao et al., 2022). Therefore, piezoelectric scaffolds can generate electrical charges in response to applied mechanical stress or deformation (Turner et al., 2001; Halperin et al., 2004; Minary-Jolandan and Yu, 2010). The stress-generated electrical signal can activate voltage-gated Ca^2+^ channels, promote displacement of C=O bonds, and favor the M2 phenotype of macrophages, leading to charge alteration on the cell membrane (Balint et al., 2014; Ud-Din and Bayat, 2014; More and Kapusetti, 2017; Murillo et al., 2017). Charge transfer promotes regenerative activities by modulating cellular behavior through molecular pathways, such as phosphatidylinositol 3-kinase (PI3K) and phosphatase and tensin homolog (Balint et al., 2014; Ud-Din and Bayat, 2014; More and Kapusetti, 2017; Murillo et al., 2017).

Representative piezoelectric scaffolds for efficient osteogenesis, including piezo-bioceramics (e.g., barium titanate (BaTiO_3_), magnesium silicate, zinc oxide) and some piezo-biopolymers (e.g., polyvinylidene fluoride, polyhydroxybutyrate, collagen, etc.), have been extensively studied (Jacob et al., 2018; Khare et al., 2020). For example, piezoelectric BaTiO_3_ was integrated into bioactive nano-titania ceramics as TiO_2_/BaTiO_3_ composite ceramics scaffolds (Li et al., 2009). BaTiO_3_ could adjust the elastic modulus of the scaffolds analogous to that of human bone (Li et al., 2009). Meanwhile, the piezoelectric properties of BaTiO_3_ also showed the enhancing effects on the bioactivity, which made the osteoblasts proliferate faster on the scaffolds *in vitro* (Li et al., 2009). Moreover, Wang’s team developed a piezoelectric poly (vinylidene fluoride-trifluoroethylene) (PVDF-TrFE) scaffold (Wang et al., 2018). Under physiological loads, the scaffold in the defect area can generate electrical potential (Wang et al., 2018). Once the osteoblasts were stimulated by the scaffold surface charges, osteoblastic proliferation would be enhanced, which promoted bone regeneration in the defect area (Wang et al., 2018). Furthermore, via electrospinning, Barbosa et al. filled PVDF-TrFE scaffolds with hydroxyapatite (HAp) (PVDF-TrFE/HAp) (Barbosa et al., 2023). Besides cell proliferation enhancement, PVDF-TrFE/HAp can boost bone tissue mineralization process and enhance the osteogenic differentiation (Barbosa et al., 2023). Also, the collagen-based composite scaffolds have been reported for efficient hard tissue engineering (Zhang et al., 2023). The compressive force on collagen triggered the re-organization of dipole moment and generated negative charges, which prompted the electrical stimulation to the cells and leads to the opening of voltage-gated Ca^2+^ channels (Zhang et al., 2023). This activity can subsequently activate the expression of osteogenesis related genes like TGFβ, BMP-2 (Zhang et al., 2023).

### 2.2 Photo-responsive composites

Photo-responsive composites are stimuli-responsive materials with minimal damage toward normal tissues and easily remote-control properties (Zhao et al., 2019a; Jamnongpak et al., 2024). When exposed to certain wavelength of light, the drug delivery ratio, shape or surface charges of photo-responsive composites may be altered in response to different categories of light (Xing et al., 2023). Lights that are commonly used for photo-responsive therapy comprise 1) visible and 2) near-infrared (NIR) lights; their wavelengths fall between 400–700 nm and 700–1,300 nm, respectively, and can penetrate most tissues to reach the target area (Escudero et al., 2019; Wan et al., 2020). For example, *in vivo* studies verified that when an 810-nm NIR light was applied to a rat’s head, 51% of the laser could transmit through the skull and 40% through the scalp and skull in the prefrontal regions (Salehpour et al., 2019).

**FIGURE 3:**
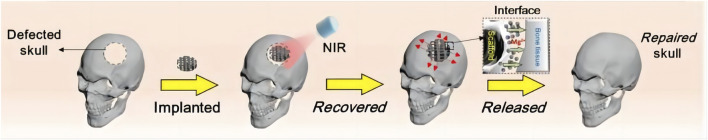
Procedure for bone regeneration using shape memory biomaterials (Reproduced with permission., Zhang et al. (2022b), Bioactive Materials).

In Zhang’s work, an NIR light-responsive scaffold that contained shape memory polyurethane (SMPU) as an SMPU/Mg composite porous scaffold was utilized (Zhang et al., 2022b). Before being implanted into the defect area, shape memory composites were programmed to a certain size (Zhang et al., 2022b). Upon exposure to NIR light, the form-programmed shape memory composites recovered, achieving tight connection with the surrounding hard tissue (Figure 3) (Zhang et al., 2022b). Accordingly, the shape memory composites precisely repaired the defective bone tissue (Zhang et al., 2022b). Moreover, Yan et al. combined graphitic carbon nitride (g-C_3_N_4_), reduced graphene oxide (rGO) with Ti-based orthopedic implant (rGO/CN/TO) (Yan et al., 2022). Under blue LED exposure, the rGO/CN/TO ternary nanocoating exhibited higher open circuit potential and transient photocurrent density (Yan et al., 2022). This exerted greater effects on enhancing osteogenic differentiation of MC3T3-E1 cells through increasing Ca^2+^ influx under visible-light stimulation (Yan et al., 2022). Therefore, the implant was proved to be able to stimulate the regeneration of bone and nerves.

### 2.3 Ultrasound-responsive composites

Ultrasound-responsive composites represent a category of stimuli-responsive biomaterials with the capacity to regulate protein release, electric charges level, structure alternation, etc., by reacting to external ultrasound radiation (Brudno and Mooney, 2015). When induced by intensity-elevated ultrasound, certain chemical linkage breaks occur, such as Diels-Alder linker, fatty acid ester bonds, and phosphoester bonds (Suslick, 2013). Thereafter, the loaded bone formation-related components are released, such as cyclooxygenase 2, prostaglandin E2, short-chain fatty acid, and dopamine-functionalized hyaluronic acid ions, which can facilitate stem cells proliferation and differentiation (Veronick et al., 2018; An et al., 2021; Arrizabalaga et al., 2022; Zhou et al., 2024). Thus, combining and modulating composites with ultrasound can enhance osteoblastic response considerably and expedite the mineralization of hard issues (Moonga and Qin, 2018; Veronick et al., 2018; Fan et al., 2020).

A myriad of multifunctional biomaterials that utilize ultrasound as stimulation to enhance osteogenesis expression were fabricated recently. Among these biomaterials, the ultrasound-responsive nanofiber hydrogel (UPN@hydrogel) has provided a novel strategy for bone repair (Zhang et al., 2024). These nanofibers can be released from hydrogel in a time-dependent manner upon ultrasound stimulation (Zhang et al., 2024). Then, nanofibers could activate M2 macrophages to secrete BMP-2 and insulin-like growth factor 1, accelerating the osteogenic differentiation of BMSCs (Zhang et al., 2024). Similarly, when targeted with focused ultrasound, crosslinking chitosan also show ability in regulating BMSCs differentiation via the breakage of innate two distinct Diels-Alder linkers and the release entrapped cytokines (Pajarinen et al., 2019; Arrizabalaga et al., 2022). Moreover, combination of ultrasound irradiation and gene-activated matrix-based therapeutics also showed promising outcomes by responsively releasing osteogenesis-related peptides, such as BMP-2/7 (Nomikou et al., 2018). In addition, with assistance of ultrasound, piezoelectric nylon-11 nanoparticles (nylon-11 NPs) could promote the osteogenic differentiation of dental pulp stem cells efficiently in a noninvasive way (Ma et al., 2019). Therefore, nylon-11 NPs are promising to be used in BTE.

### 2.4 Magneto-responsive composites

Lately, researchers have integrated magnetic nanoparticles, such as Co_3_O_4_, Fe_3_O_4_ or MnFe_3_O_4_ nanoparticles, into various matrices to fabricate magnetic composites, exploring the potential for application as bone scaffolds or substitutes (Xu and Gu, 2014; Lui et al., 2019). When subjected to an external magnetic field, magneto-responsive nanocomposites exhibit magnetic twisting or clustering responsiveness and can function as carriers for biologically or chemically active agents via magneto-driven delivery (Cojocaru et al., 2022). By delivering bone-forming substances, such as BMP-2 and dexamethasone acetate, magneto-responsive nanocomposites are conducive for bone regeneration (Butoescu et al., 2009; Wang et al., 2012).

Magneto-responsive nanocomposites allow several processing options. Tang et al. designed a magneto-responsive CoFe_2_O_4_/P(VDF-TrFE) nanocomposite (Tang et al., 2021). Cellular osteogenic differentiation can be enhanced when the nanocomposite is exposed to magnetic field. Moreover, this material can significantly upregulate the expression level of α2β1 integrin and p-ERK, which exhibited promising potential in bone tissue repair and regeneration. Meanwhile, Wu et al. fabricated a ceramic composite containing super-paramagnetic nanoparticles (Wu et al., 2010). *In vivo* experiments demonstrated that the super-paramagnetic nanoparticles integrated in the composites accelerated new bone-like tissue formation (Wu et al., 2010).

Magnetic Resonance Imaging (MRI) is widely used for clinical examinations. It is a common intermittent pulsing electromagnetic field. When magneto-responsive nanocomposites are exposed to MRI, the stiffness of magneto-responsive nanocomposites increases due to the rearrangement of magnetic particles (Li et al., 2020). Then, integrins on the stem cell membrane transferred information about the mechanical state of the extracellular matrix into cells (Li et al., 2020). Activating osteogenic differentiation signal pathways, such as the PI3K/Akt pathway, enhances stiffness, increases the number of mitochondria, and changes cell morphology (Yan et al., 1998; Lambertini et al., 2015; Androjna et al., 2021). These results suggest that stem cell osteogenic differentiation can be regulated; MRI appears to positively affect magneto-responsive nanocomposites when used for patient examinations.

External stimuli-responsive biomaterials permit noninvasive activation and remote control (Yang et al., 2018; Lin et al., 2020; Wan et al., 2020; Zhao et al., 2020). In addition, targeted drug delivery to specific sites within the body can be achieved via out-body stimulation such as magnetic guidance (Lu et al., 2018; Lui et al., 2019). However, it is challenging to achieve optimal performance and responses. Moreover, external stimuli, such as NIR light and ultrasound, may generate heat in the tissue, leading to thermal damage (Sun D. et al., 2020; Arrizabalaga et al., 2022). Therefore, further studies are needed to improve this type of biomaterials. More details on the advantages and disadvantages of stimuli-responsive biomaterials are listed in Table 1.

**TABLE 1:** Advantages and disadvantages of different stimuli-responsive biomaterials.

	Biomaterial	Advantages	Disadvantages	References
External stimuli-responsive biomaterials	Piezoelectricity-responsive scaffolds	1) Mechano-electrical coupling can mimic natural processes in the body where mechanical stimuli influence cellular behavior 2) The dual functionality of stress responsiveness and piezoelectricity may enhance cellular response to the scaffold	1) It is challenging to achieve optimal performance and responses 2) Studies on piezoelectric biomaterials in the repair and regeneration of hard tissues remain limited	Jacob et al. (2018), Tandon et al. (2018), Ghosh et al. (2022)
	NIR light-responsive 3D-printed shape memory composites	1) NIR light permits the noninvasive activation of the scaffold within the body 2) NIR light-responsive scaffolds can be engineered with various functionalities, such as drug release, photothermal effects, or changes in material properties, offering versatility in their applications	1) Prolonged exposure to NIR light can lead to photobleaching of the photosensitive components in the scaffold, potentially reducing their responsiveness over time 2) The depth of penetration remains limited	Yang et al. (2018), Lin et al. (2020), Wan et al. (2020), Zhao et al. (2020)
	Ultrasound-responsive hydrogels	1) Ultrasound allows for precise control over the location and timing of hydrogel activation 2) Ultrasound can be applied externally, providing remote control for activating the hydrogels	1) High-intensity ultrasound may generate heat in tissues, potentially leading to thermal damage 2) The depth of penetration is limited	Sun et al. (2020a), Arrizabalaga et al. (2022)
	Magneto-responsive nanocomposites	Targeted drug delivery to specific sites within the body can be achieved via magnetic guidance	May present biocompatibility challenges	Lu et al. (2018), Lui et al. (2019)
Internal stimuli-responsive biomaterials	pH-responsive hydrogels	1) The release of drugs or therapeutic agents from pH-responsive hydrogels can be finely controlled by pH levels 2) Can be engineered to respond to a wide range of pH values	pH changes in the body may occur in response to various factors, and unintended activation of pH-responsive hydrogels could lead to undesirable consequences	Chen et al. (2019), Zhang et al. (2020), Montoya et al. (2021)
	Thermo-responsivehydrogels	1) The state of hydrogel can be altered and easy to be injected 2) Injectable thermo-responsive hydrogels can fit into the shape of bone defect integrally	Temperature alteration cannot be controlled precisely	Yu et al. (2021), Kim et al. (2023)
	ROS-responsive scaffolds	1) Can be tailored for disease-specific activation and intervention 2) Can deliver therapeutic agents precisely in response to the pathological conditions	Reactive oxygen species generation or consumption by the scaffold may result in oxidative stress	Hayyan et al. (2016), Sun et al. (2020b), Ren et al. (2022)
	Enzyme-responsive scaffolds	1) Enable precise control over the release of therapeutic agents 2) Can be designed to mimic and interact with natural biological processes, which facilitate integration with the body’s existing systems and enhances compatibility	1) Achieving a balance between sufficient stability for the scaffold and appropriate biodegradability can be challenging 2) The response to an enzymatic stimulus can in some cases depend on the age of the host	Boskey and Coleman (2010), Nguyen et al. (2018), Berillo et al. (2021)
	Ion-responsive scaffolds	Possess stable structure, low cytotoxicity, great specific surface area and versatile usage	Relating researches are limited	Simon-Yarza et al. (2018), Gao et al. (2019), Kumar et al. (2021)
	Glucose-responsive biomaterials	Effectively utilize superfluous glucose in blood to renovate the oppressed bone remodeling.	The effectiveness of osteogenesis promotion via blood sugar is not yet clear.	(Mohanty et al., 2022)

## 3 Biomaterials responding to internal stimuli

**FIGURE 4:**
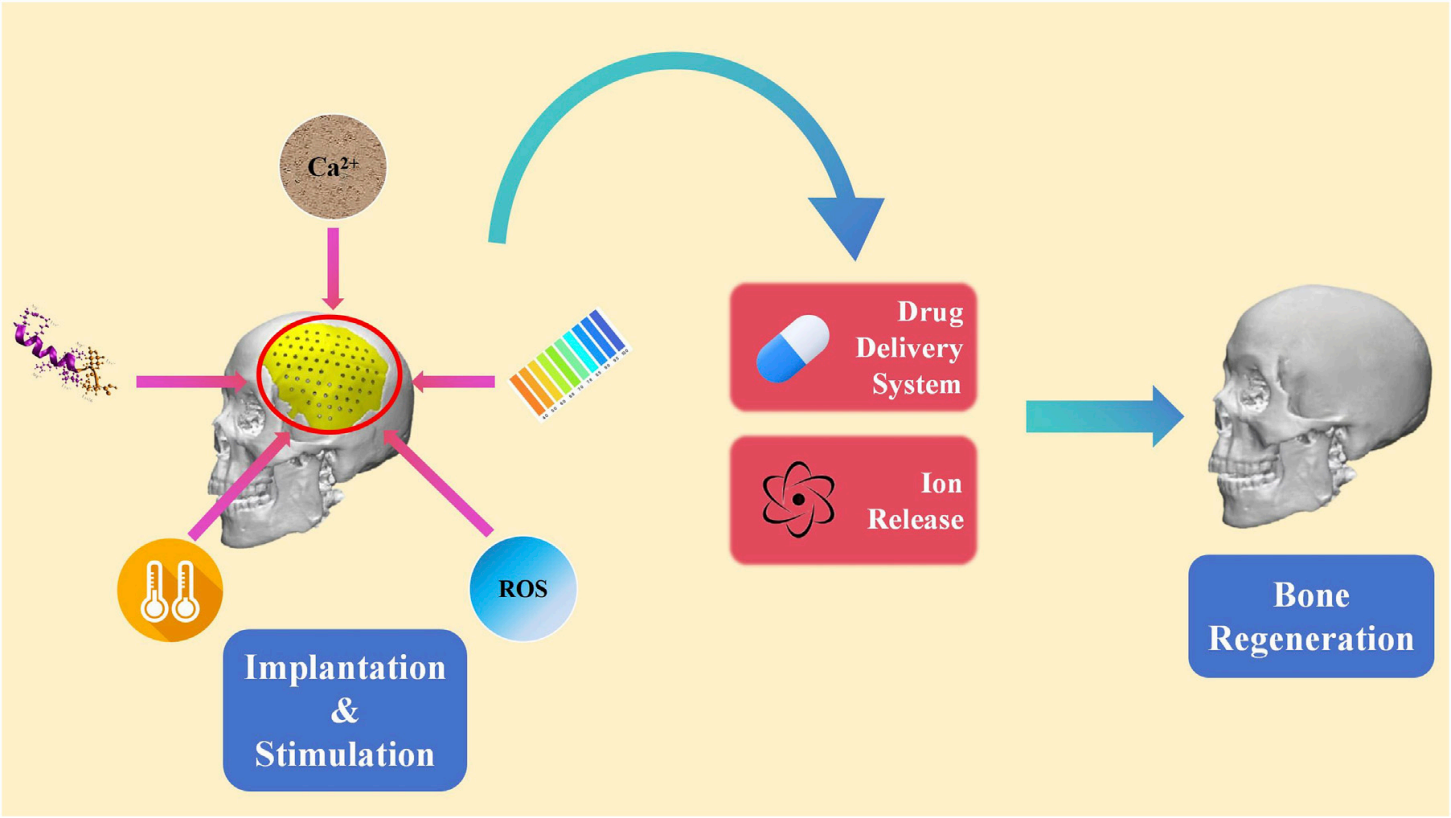
Schematics of internal stimuli-responsive biomaterials.

Internal or in-body stimulus refers to signals in the microenvironment inside the body around the biomaterial (Lee et al., 2018a; Nguyen et al., 2018; Yao et al., 2019; Ding et al., 2022). These stimuli comprise of pH, temperature, ROS, enzyme ion concentration and etc. Drug carriers of internal stimulus-responsive biomaterials can be designed to respond to specific triggers, such as pH changes, ROS fluctuations, or the presence of specific biomolecules. Thus, internal stimulus-responsive biomaterials are able to deliver the loaded medicine to the target area or release certain ions when the concentration of the chemical substance changes (Figure 4) (Hein et al., 2008; Lallana et al., 2012; McKay and Finn, 2014; Gregoritza and Brandl, 2015). Furthermore, the released medicine can exert an impact on the metabolism of a series of osteocytes. This level of precision minimizes the side effects and enhances the overall efficacy of the treatment. To develop a direct approach for bone regeneration, various studies focusing on internal-responsive biomaterials have been performed.

### 3.1 pH-responsive composites

The pathological circumstance, such as tumor or inflammation, is mildly acidic, whereas the physiological pH is neutral (7.0-7.4) (Swietach et al., 2014). pH-responsive composites have the capacity of reacting to specific pH levels in physiological and pathological environments (Li et al., 2023; Xu et al., 2023; Zheng et al., 2023). The composites have pH-sensitive coordination bonds, such as catechins, ferric irons, citraconic amide bonds, and Schiff base bonds. These bonds can change with the fluctuation in pH levels (Liang et al., 2021; Jo et al., 2023). Therefore, the state of composites can alter accordingly (Kocak et al., 2020; Zhu et al., 2021). Drugs related to BTE can be delivered by alteration between different states (Zhu et al., 2018; Constantin et al., 2020; Ding et al., 2022). When sensing the change in pH levels, these composites can set up targeted and localized drug delivery (Liu et al., 2017). Finally, certain drugs are able to upregulate the expression of Runx2, osterix, OCN, and OPN and support mesenchymal stem cells proliferation, adhesion, osteoinduction, and biocompatibility, which are essential to regenerate bone tissues (Lavanya et al., 2020).

**FIGURE 5:**
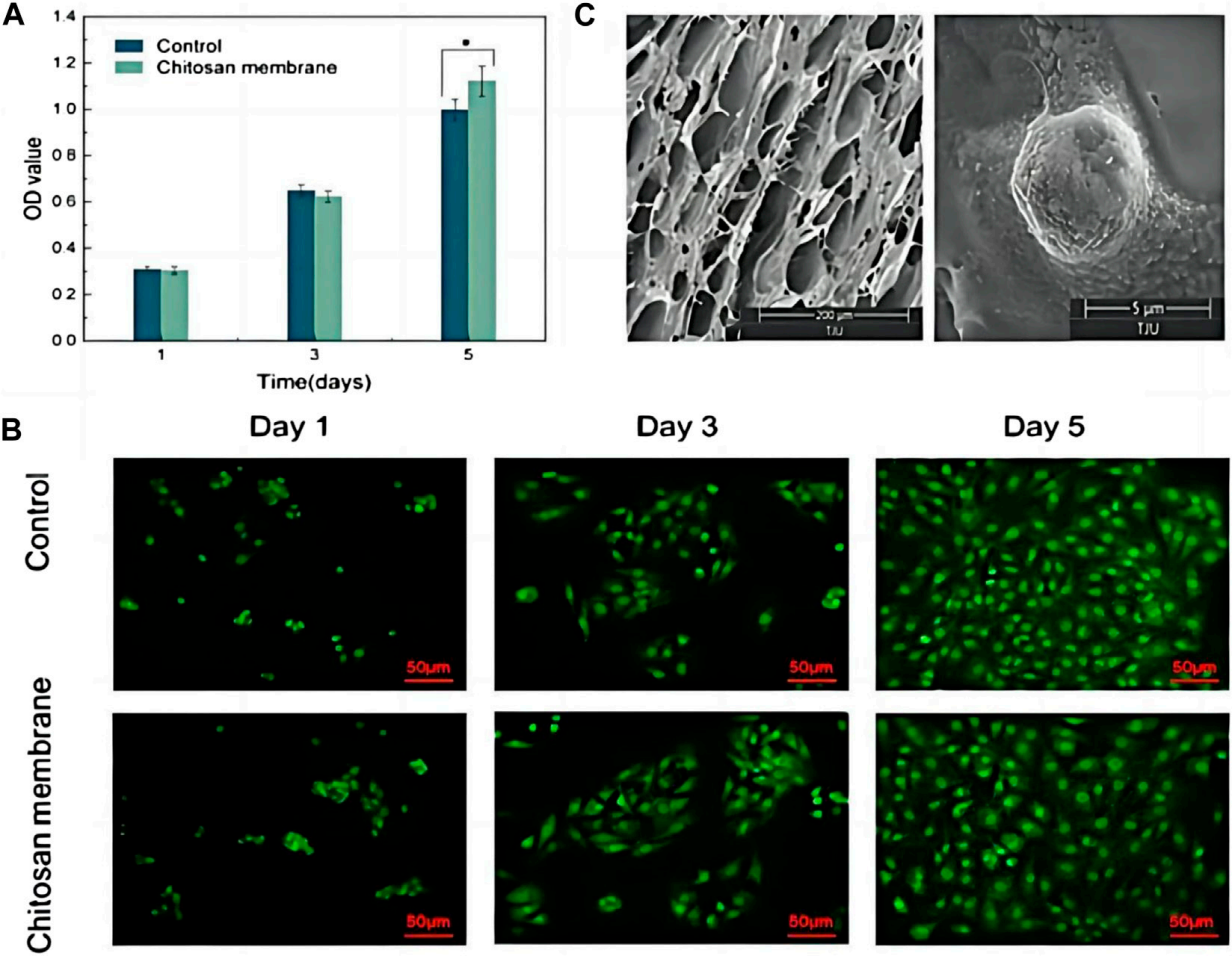
**(A)** CCK-8 assay of osteoblasts seeded into wells (Control) and on the surface of the chitosan membrane. *p < 0.05. **(B)** LSCM images of osteoblasts in wells and attached to the chitosan surface. Scale bar in LSCM images is 50 µm. **(C)** SEM images of the surface of chitosan membrane in 200 µm scale (left), and osteoblasts attached on the chitosan surface after 5 days culturing in 5 µm scale (right). Reproduced with permission., Chen et al. (2023), MDPI.

To fabricate composites with pH-responsive drug release property, Tang et al. designed a hydrogel with a alendronate-modified oxidized alginate network (GelMA-OSA@ALNDN hydrogel) in which Schiff base bonds were distributed uniformly (Tang et al., 2022). Alendronate (ALN) is a type of bisphosphonate with promising hard tissue repair functions (Wang et al., 2021). Therefore, by reacting to changing pH level, GelMA-OSA@ALNDN hydrogel can maintain a stable drug concentration to activate the repair process in the defective bone area (Tang et al., 2022). Besides, an asymmetric microfluidic/chitosan hydrogel, poly [2-(dimethylamino) ethyl methacrylate] (PDMAEMA) hydrogel, was successfully fabricated (Chen et al., 2023). The hydrogel was demonstrated with the ability of achieving pH-responsive drug release to promote osteoblast proliferation and combating with bacterial infection simultaneously (Figure 5) (Chen et al., 2023).

Currently, researchers tend to synthetize CS-based biomaterials, which are natural polymers with enzymatic biodegradability, pH sensitivity, polycationic nature, extensive application, etc. (Almajidi et al., 2023). For example, pH-responsive carboxymethyl chitosan/amorphous calcium phosphate (CMC-ACP) hydrogel system was designed (Zhao et al., 2019b). This hydrogel system was found to significantly upregulate the expression of bone markers, such as Runx2, osterix, OCN, and OPN (Zhao et al., 2019b). *In vivo* findings also showed that the injectable hydrogel strongly enhances the efficiency and maturity of the bone regeneration while suppressing the bone resorption (Zhao et al., 2019b). Furthermore, Ressler et al. synthesized a pH-responsive chitosan-hydroxyapatite hydrogel (CS/HAp/NaHCO_3_) (Ressler et al., 2018). Activating by acidic microenvironment, CS/HAp/NaHCO_3_ can improve stability, homogenous dispersion of mesenchymal stem cell (MSCs) and promote calcium phosphate deposits and extracellular matrix (ECM) mineralization (Ressler et al., 2018).

### 3.2 Thermo-responsive composites

Phase transition temperature refers to the temperature required to induce a change between phases, such as solid and liquid, sol and gel (Scott, 1974). Thermo-responsive composites exhibit reversible phase transition effect in response to change in temperature (Kim and Matsunaga, 2017). The rapid shift from a sol to a gel state at physiologic temperature can optimize the release of loaded TGF-β, drugs or ions, which has positive effect in boosting osteoblasts adherence, differentiation and proliferation (Duan et al., 2020; Khan et al., 2022).

To address the issue of the efficient local delivery of BMP-2 to complex bone fracture sites, BMP-2-functionalized MgFe-layered double hydroxide nanosheets were integrated into chitosan/silk fibroin hydrogels (CSP-LB hydrogel) (Lv et al., 2023). This thermo-responsive hydrogel solution can be injected to fit the defects precisely and then solidify quickly under 37°C condition (Lv et al., 2023). The solidified hydrogel showed 4.5-fold bone volume and 3.6-fold bone mineral density increment compared with that of the control group (Lv et al., 2023). Simultaneously, CS thermogels, supported with demineralized bone matrix, could retain more cells and provide better strength for efficient chondrogenesis in both *in vitro* and *in vivo* (Huang et al., 2014). Wu et al. constructed a thermo-sensitive N-isopropylacrylamide-chitosan hydrogel (Wu et al., 2018). This hydrogel ensured osteoinduction and biocompatibility (Wu et al., 2018). And these properties were proved by *in vitro* tests with infrapatellar fat pad-derived mesenchymal stem cells (IFP-MSCs), fibroblasts (NIH-3T3), and osteoblasts (MC3T3-E1) (Wu et al., 2018).

### 3.3 ROS-responsive composites

**FIGURE 6:**
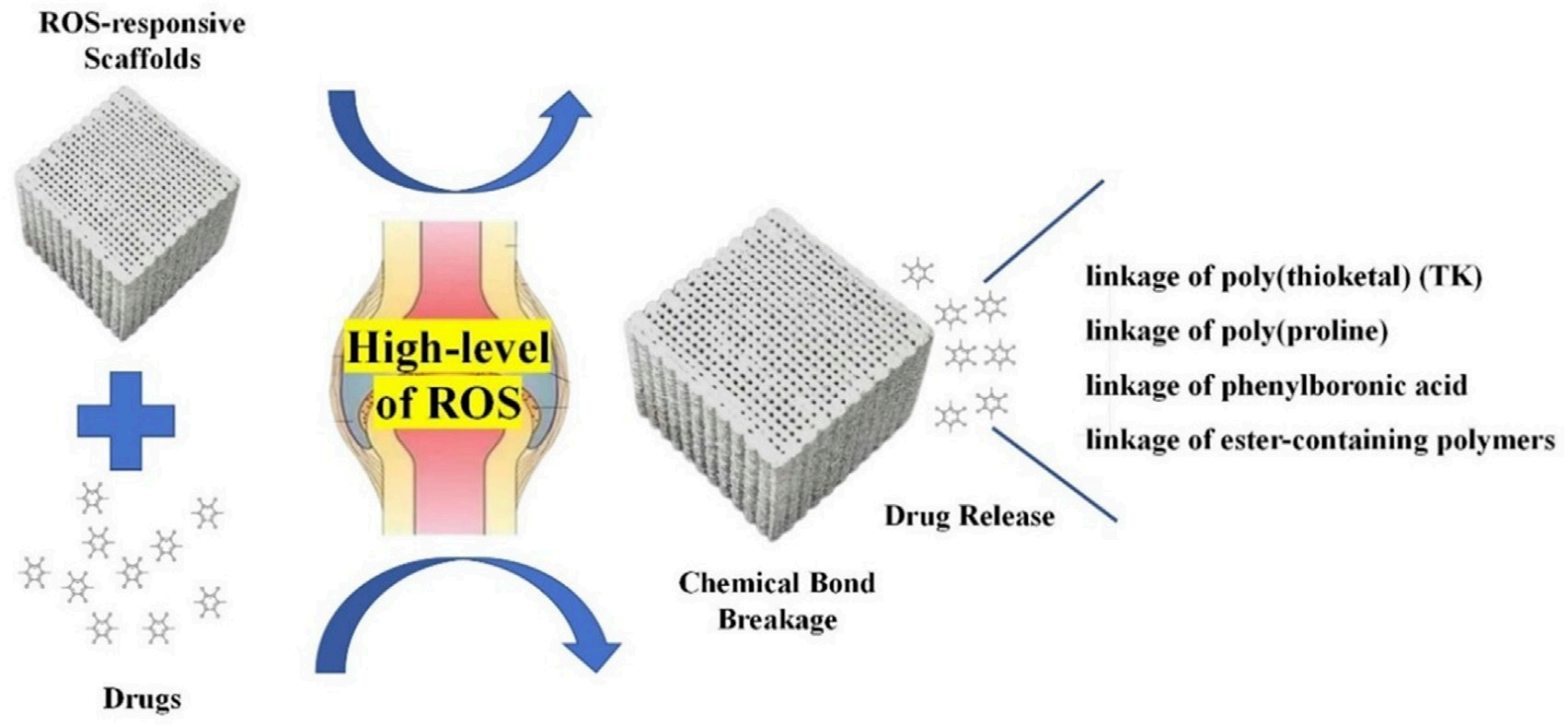
Unique features of ROS-responsive biomaterials under oxidative conditions.

ROS are a family of reactive chemical species that contain oxygen (Zorov et al., 2014). ROS have been considered pivotal signaling molecules in many physiological processes and are usually overproduced in various inflammatory tissues (Yao et al., 2019). These molecules can influence the differentiation of osteoclasts (Badila et al., 2022). ROS-responsive composites are a series of biomaterials that can target the high-level ROS in bone-related diseases (Ren et al., 2022). These scaffolds possess unique features under oxidative conditions (Figure 6) and can fit in different bone defects to achieve bone regeneration (Lee et al., 2013; Xu et al., 2016; Ren et al., 2022). Moreover, these materials can support osteocyte adhesion and growth owing to their porous structures to boost the BTE (Yao et al., 2019).

**FIGURE 7:**
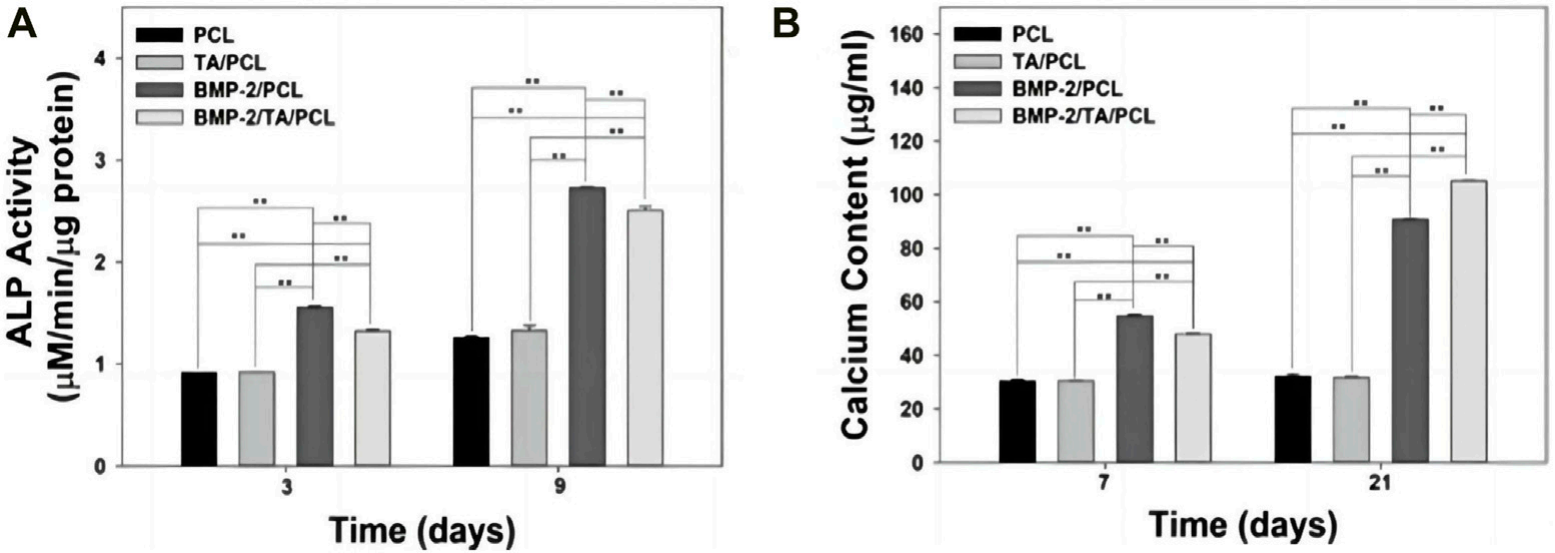
**(A)** Alkaline phosphatase activity and **(B)** calcium deposition of MC3T3-E1 cells cultured on PCL, TA/PCL, BMP-2/PCL, and BMP-2/TA/PCL scaffolds. Error bars represent mean ± SD. Reproduced with permission., Yang et al. (2020), MDPI.

In order to renovate the adverse effect of ROS stimulation to bone regeneration, Martin et al. synthesized a layer-by-layer-compatible polycation (Yamaguchi et al., 1991). This polycation presented an ROS dose-dependent delivery of the preloaded BMP-2 and promoted the cellular proliferation of progenitor cells and spurred stem cell differentiation into bone-forming osteoblasts (Yamaguchi et al., 1991; Tian et al., 2022). Moreover, a 3D-printed ROS-responsive molybdenum (Mo)-containing bioactive glass ceramic scaffold was developed recently (Lee et al., 2018b). The scaffold decreased mitochondrial ROS produced by osteoclasts and increased the expressions of certain genes related to osteogenesis, such as matrix metalloprotein (MMP), NFATc1, and RANKL (Lee et al., 2018b). In the study by Lee et al., 3-dimension polycaprolactone (PCL) scaffold with tannic acid (TA) and BMP-2 (BMP-2/TA/PCL) was fabricated (Yang et al., 2020). In the ROS-overproduction environment, the BMP-2/TA/PCL scaffold maintained the sustained and controlled release of BMP-2, which stimulated the osteogenic differentiation of MC3T3-E1 cells by increasing ALP activity and calcium deposition (Figure 7) (Yang et al., 2020).

### 3.4 Enzyme-responsive composites

Enzymes play pivotal roles in growth, blood coagulation, wound healing, respiration, digestion, and various other physiological processes (Yang et al., 2020). Disruptions in the expressions or activities of enzymes lead to severe pathological conditions, including but not limited to cancer, cardiovascular disorders, inflammation, and degenerative arthritis (Hu et al., 2014). Composites responsive to enzymes are activated by the selective catalytic activities of specific enzymes (Zhang et al., 2016). For example, type II collagen forms the base for the formation of cartilage and bone (Anjum et al., 2016; Ding et al., 2020; Fischer et al., 2021). Local gene expression and the secretion of type II collagen can be regulated by the overexpression of enzymes such as polygalacturonase, BMPs, and matrix metalloprotein (Anjum et al., 2016; Ding et al., 2020; Fischer et al., 2021).

Blood coagulation factor XIII (FXIIIa) is closely related to bone tissue repair. This enzyme can regulate the RANKL/RANK system in MSCs, augmenting osteoblast differentiation and mineralization (Ikebuchi et al., 2018; Dang et al., 2023). Therefore, Anjum et al. built an enzymatically formed chondroitin sulfate and poly (ethylene glycol) (PEG)-based hybrid hydrogel scaffold system (Anjum et al., 2016). Induced by FXIIIa-catalyzed glutaminating reaction, the degraded hydrogel released BMP-2, facilitating the integration of the newly formed bone tissue (Anjum et al., 2016).

### 3.5 Ion-responsive scaffolds

Inbody ion level may shift in some physiological and pathological environment (Mészáros et al., 2022; Ye et al., 2022; Adella and de Baaij, 2023). Therefore, the fluctuation of ions concentration like Na^+^, K^+^, Ca^2+^ can be used as the internal stimulation to activate the ion-responsive scaffolds (Zhou et al., 2013; Xu et al., 2015). Certain ions loaded on the porous surface of the ion-responsive scaffolds would be displaced when the materials were activated. The released ions could automatically combine with hydroxy phosphates (Wang et al., 2023). The self-combination results in large hydroxyapatite-like crystals *in vivo*, accelerating bone remineralization and regeneration (Zhou et al., 2013; Gao et al., 2021; Wang et al., 2023).

Sun et al. used melt electrowritten printing technology to design a mineralized zeolitic imidazolate framework-8/polycaprolactone (ZIF-8/PCL) scaffold (Wang et al., 2023). In a simulated body fluid solution, Zn-N bonds and hydrogen bonds in ZIF-8 slightly decomposed because of the competitive binding of Ca ions, forming excess insoluble zinc hydroxy phosphates (Wang et al., 2023). Apatite and zinc positively promote bone regeneration by rising the biocompatibility of biomaterials and promoting tissue integration (Su et al., 2019; Gao et al., 2021). Accordingly, ZIF-8 showed a favorable impact on promoting osteogenesis. Moreover, by mixing ZIF-8 and PCL, the scaffold presented low inflammatory responses and increased biocompatibility (Wang et al., 2023).

### 3.6 Glucose-responsive biomaterials

Diabetes mellitus (DM) is an intricate disorder of glucose metabolism. The incidence of DM is projected to reach 10.2% by 2030 and 10.9% by 2045 (Saeedi et al., 2019). The irregular hyperglycemic environment makes the cells and tissues in dysfunction and hinder the osseointegration process (Khosla et al., 2021). Hence, biomaterials can effectively utilize superfluous glucose in blood to renovate the oppressed bone remodeling are fairly in need. And series glucose-responsive biomaterials have emerged. For instance, a functional glucose-responsive immunomodulation-assisted periosteal regeneration composite material, Polylactic Acid/Collagen I/Liposome-APY29 (PCLA), was constructed (Qiao et al., 2023). In the DM microenvironment, the high glucose can promote the combination of hydroxyl groups grafted in the glucose-responsive composites (Mohanty et al., 2022; Qiao et al., 2023). This makes the composite surface changes from hydrophobic to hydrophilic, which is beneficial to promote cell adhesion and proliferation (Mohanty et al., 2022; Qiao et al., 2023). Meanwhile, by blocking the IREα/NOD-like/NF-κB signaling pathway, PCLA can remodel the pathologic diabetic microenvironment into a regenerative microenvironment (Qiao et al., 2023). Moreover, He et al. devised a glucose-primed orthopedic polyetheretherketone (PEEK) implant (sP-P@C-G) composed of Cu-chelated metal-polyphenol network (hauberk coating) and glucose oxidase (GOx) (He et al., 2023). In hyperglycemic microenvironment, glucose-responsive enzymatic cascade can be activated by GOx to produce endogenous H_2_O_2_ (He et al., 2023). Then, dopamine (DA) anchor onto PEEK implant surfaces in the alkaline environment via self-polymerization (He et al., 2023). DA shows promising ability on facilitating cell attachment, which endows sP-P@C-G with appealing biocompatibility and outstanding osteogenicity (Chen et al., 2017; He et al., 2023).

When compared with external stimuli-responsive biomaterials, the primary benefit of internal stimuli-responsive biomaterials lies in their capacity for activation without external stimuli (Berillo et al., 2021). These materials may lower the cost of purchasing certain external machines and alleviate the workload of treatment (Boskey and Coleman, 2010). However, changes in the specific stimuli may happen under various conditions and may lead to the unintended activation of the materials (Chen et al., 2019; Zhang et al., 2020; Montoya et al., 2021). In addition, achieving a balance between sufficient stability of the materials and appropriate biodegradability can be challenging (Boskey and Coleman, 2010; Nguyen et al., 2018; Berillo et al., 2021).

## 4 Conclusion

Stimuli-responsive biomaterials have been considerably explored and have garnered substantial attention. As the interdisciplinary collaboration among materials science, biology, and medicine continues to flourish, the future holds exciting prospects for stimuli-responsive biomaterials. Nonetheless, despite the promising bone regeneration effect, stimuli-responsive biomaterials are still in infancy with challenges need to be settled:

1) Due to insufficient sample size, inadequate simulated conditions and deficient number of experiments, the clinical application evidence for most stimuli-responsive biomaterials is still limited. Therefore, more high-quality studies and preclinical studies are needed.
2) More and more studies tend to design multiple stimulation-responsive biomaterials. However, it remains to be discussed that whether applying two or more stimulus would obtain better bone regeneration effect than single one.
3) Proper application conditions for valid osteogenesis need to be verified. For example, feasible pH stimulation range for optimal bone regeneration effect, suitable magnetic field strength for BTE, etc.
4) Current synthetic processes of stimuli-responsive biomaterials are generally time-consuming and complicated. Thus, the exploration of safer and more efficient synthetic processes is necessary.

In a nutshell, although certain challenges persist and clinical translation remains a formidable task, it is conceivable that the integration of intelligent stimuli-responsive materials holds considerable promise for transformative biomedical applications in the future. Consequently, there is still a long way to go to reach the optimum of ideal stimuli-responsive biomaterials for bone regeneration.
